# Putative mechanisms of caffeine as a neuroprotectant in preterm infants

**DOI:** 10.1038/s41390-026-04776-0

**Published:** 2026-01-15

**Authors:** Madeline A. MacNamara, Paul B. Colditz, Julie A. Wixey

**Affiliations:** 1 UQ Centre for Clinical Research, Faculty of Health, Medicine and Behavioural Sciences, The University of Queensland, Brisbane, QLD Australia; 2 Perinatal Research Centre, Royal Brisbane and Women’s Hospital, Brisbane, QLD Australia

## Abstract

**Abstract:**

Survival rates of preterm infants have been steadily increasing over the last few decades. This has drawn long-term outcomes in this population to the forefront of scientific attention. Caffeine is a pharmacological agent that is available to neonatologists in the treatment of apnoea of prematurity. Its use for this condition has been well researched; however, less focus has been directed towards its neuroprotective capabilities. Long-term follow-up from the landmark ‘Caffeine Therapy for Apnoea of Prematurity’ trial suggested that caffeine may confer benefits in neurodevelopmental outcomes. The indirect ways in which caffeine could confer these benefits have been extensively discussed; however, the direct molecular mechanisms by which this is achieved are poorly understood. This article reviews the putative mechanisms through which caffeine acts as a neuroprotectant in the preterm infant. Caffeine may directly confer neuroprotective benefits through altering white matter structures, reducing inflammation, preventing cell death and conferring neuromodulatory benefits. With the rise in preterm birth and the need for effective neuroprotective therapies, caffeine’s multifaceted mechanisms offer promising avenues for future research and clinical application.

**Impact:**

- This article adds to the existing literature by which it: reviews the putative mechanisms through which caffeine acts as a neuroprotectant
- hypotheses that caffeine confers neuroprotective benefits through altering white matter structures, reducing inflammation, preventing cell death, and conferring neuromodulatory benefits, and
- consolidates evidence regarding caffeine’s impact on neurodevelopmental outcomes

## Introduction

The World Health Organisation (WHO) defines a preterm birth as any occurring before 37 weeks of gestation, and a very preterm birth as occurring before 32 weeks.^1^ Infants born with a low birth weight (LBW), either as a result of intrauterine growth restriction, prematurity, or both, are also an important population to consider in neonatology, defined as children with a birth weight <2500 g or as very low birth weight (VLBW) when weighing <1500 g.^2^ Both LBW and preterm infants comprise a heterogeneous group with a broad range of health and neurodevelopmental outcomes.^3,4^ Mortality rates have been decreasing recently for preterm and LBW infants, shifting focus towards promoting long-term outcomes.^5,6^ An important long-term consideration is neurodevelopment, which is significantly more likely to be poor in very preterm and VLBW children.^7,8^ Despite significant improvements in survival rates, an equivalent improvement in long-term neurodevelopmental outcomes has not occurred.^9^

There is often debate and controversy surrounding neuroprotective strategies in preterm infants, with long-term efficacy uncertain for many available treatments.^10^ Although maternal corticosteroids and magnesium sulphate may have beneficial effects on the developing preterm brain, it is not always possible to administer these therapies to mothers expecting preterm birth. This could be due to unexpected preterm birth or access to medication in low-income countries. A vital area is to develop safe and effective postnatal neuroprotective interventions, increasingly important as the number of premature children with neurodisability is increasing.^10^ Caffeine is a commonly used medication in the management of apnoea of prematurity (AOP).^11^ This medication is safe in preterm and LBW infants, is inexpensive, convenient to administer, and easy to monitor for side effects.^12–15^ Although evidence exists to suggest that caffeine may have a neuroprotective effect in preterm and LBW infants, there is a paucity of analysis regarding the mechanisms by which this could be achieved.^9,16^

Currently, the neuroprotective mechanisms of caffeine remain unclear. Historically, it was thought that the susceptibility of this population to traumatic and macroscopic brain injury was responsible for preterm neurodevelopmental vulnerability.^9^ The Caffeine for Apnoea of Prematurity (CAP) trial challenged this belief, finding that caffeine was associated with long-term neurodevelopmental benefits, despite ultrasonographic findings that types and frequency of brain injury were similar between groups.^17^ The idea that caffeine confers neurodevelopmental benefits through both direct and indirect mechanisms is now a generally accepted concept.^18^ Multiple direct mechanisms have been proposed in the literature hypothesising that caffeine has pro-myelin, anti-inflammatory, anti-apoptotic, and neuromodulatory properties. This review seeks to present a synthesised summary of the current evidence behind these proposed mechanisms. Ultimately, the aim of this study is to provide further clarity on caffeine’s role as a neuroprotectant.

## Methods

A search was conducted using PubMed, Embase, and Web of Science to identify studies for this narrative review, which were published up to September 2025. Search terms included ‘caffeine’, ‘neuroprotection’, ‘preterm’ and ’neurodevelopment’. Reference lists were also screened to identify further eligible studies. We have defined ‘neuroprotectant’ broadly as an agent that promotes or facilitates normal neurodevelopment by whatever mechanism and regardless of whether the target cell is neuron, glial cell, oligodendrocyte, or inflammatory pathways involving multiple cell types.

### Neurodevelopment in preterm infants

Complications from preterm births are the leading cause of death in children under the age of 5 years.^19^ As such, preterm infants are a well-studied population with improving survival rates shifting scientific interest further towards long-term outcomes.^5,6^ Neurodevelopmental outcomes for both preterm and LBW infants are poor,^5^ with these infants more likely to be diagnosed with cerebral palsy, cognitive impairment, developmental delay and behavioural disorders than term infants.^20–24^ School achievement requires competence in physical function, emotional skills, communication, and cognitive and executive function.^25^ Almost half of children born extremely preterm experience academic and behavioural difficulties at school.^8^ Furthermore, children born preterm or LBW are more likely to experience significant motor impairment that persists throughout childhood.^7^ Motor dysfunction that significantly interferes with a child’s life and is not associated with a general medical condition is diagnosed as Developmental Coordination Disorder (DCD).^26,27^ This condition occurs more frequently in preterm and LBW populations and is associated with poor outcomes in multiple domains: cognition, academic performance, behavioural development, attention, learning, psychosocial adjustment, and somatic complaints.^28–30^ Currently, there are no effective preventative measures or treatments for DCD.^27^

Preterm infants are subject to a host of unique factors that predispose them to these poor neurodevelopmental outcomes. The preterm brain is immature in its development, making it vulnerable to three major forms of brain injury: germinal matrix-intraventricular haemorrhage, cerebellar haemorrhage and white-matter injury.^31^ Furthermore, it has historically been recognised that impaired brain development contributes to the poor neurodevelopmental outcomes seen in preterm infants.^31–33^ This process, an initial insult and the adverse effect it has on the development of grey and white matter, is referred to as dysmaturation. It is thought that the poor neurodevelopmental outcomes seen in preterm infants are a consequence of a variety of dysmaturational disturbances in the brain.^31,34^ The dysmaturational causes include hypoxia, infection/inflammation, nutrition, and environmental factors. These processes are far-reaching and can include insults to myelination, axonal development, and the distribution of activated microglia and reactive astrocytes.^34^ The current postnatal neuroprotective interventions that exist for preterm infants have limited efficacy.^31,34^ As a consequence, there is a growing interest in neuroprotective interventions that are both safe and improve long-term neurodevelopmental outcomes for preterm neonates.

### Caffeine for the preterm infant

Caffeine citrate has been a mainstay of treatment for AOP over the last four decades, with first reported use in 1977.^35,36^ AOP is defined as a cessation of breathing that occurs for more than 15 s and is accompanied by hypoxia or bradycardia.^37^ This condition is common in preterm infants, occurring in 85% of infants born at less than 34 weeks of gestation.^37^ The use of caffeine to treat this condition has become an internationally widespread practice as it reduces apnoeic episodes through increasing minute ventilation, CO_2_ sensitivity and central nervous system respiratory drive.^11,38^

Caffeine is a methylxanthine, a group of non-selective antagonists of adenosine receptors that includes aminophylline, theophylline and caffeine.^13,38^ Adenosine receptors are found in moderate concentrations in the lungs and in high concentrations in the brain.^39^ The main receptors that caffeine affects are the A1 (A1R) and A2A (A2AR) adenosine receptors—G-protein coupled receptors with opposing actions of inhibiting and stimulating adenyl cyclase (AC), respectively.^40^ These receptors are located on presynaptic terminals and act primarily in the cerebral cortex and hippocampus (A1R) and the striatum (A2AR), decreasing glutamate release and increasing dopamine release, respectively.^41^ The pharmacokinetics of caffeine have been extensively researched and reveal a compound that is both safe and convenient for use in preterm infants.^42^ Caffeine offers a wide therapeutic range and does not require routine blood concentration measurements to assess toxicity.^13,14^ It has a long serum half-life and can last up to 101 h in the neonatal system before being metabolised in the liver and cleared renally.^43–45^ Caffeine is a cost-effective medication that is generally well tolerated by infants at standard doses.^12,46,47^ Reports of adverse effects are infrequent but include tachycardia, feed intolerance, vomiting, glucose instability, tachypnoea, tremor and irritability.^14,47^

Interest in caffeine peaked with the release of the landmark CAP trial in 2006. This randomised controlled trial found that caffeine reduced rates of bronchopulmonary dysplasia (BPD) and mechanical ventilation use in VLBW infants.^15^ This is pertinent as there is a demonstrated link between BPD severity and adverse neurodevelopmental outcomes.^48^ A follow-up study of these infants at 18–21 months found that the caffeine group had significantly lower rates of neurodevelopmental disability.^49^ At 5 and 11 years follow-up, it was noted that these benefits had only lasted for motor function, with lower rates of DCD in the caffeine group compared to placebo.^17,49,50^ Thus, interest was directed towards caffeine’s potential as a neuroprotectant in preterm infants. Demonstration of the association between caffeine and improved neurodevelopmental outcomes has also been shown in multiple domains (not only motor), independent of the severity of lung disease.^51,52^ Caffeine has well-established neuroprotective effects in adults but is not traditionally considered as a neuroprotective intervention for preterm infants.^31,34,40,53^ Caffeine is shown to be neuroprotective in Alzheimer’s disease and Parkinson’s disease at dosages equivalent to 3–5 mg/kg.^53^ However, caffeine dose for the preterm infant can be much higher (see Table 1). There is some recognition of its influence over neurodevelopment through preventing BPD, a risk factor for neurosensory issues; however, the intrinsic qualities of caffeine in preserving neural structures are rarely considered.^34,54^ As evidence suggests that caffeine provides neurodevelopmental benefits in a population for which few neuroprotective interventions exist, it is important that these intrinsic qualities are discussed.^31^

**Table 1:** Summary of human clinical trials analysing caffeine’s effect on neurodevelopmental outcomes in preterm infants.

Study	Study type	Sample size	Population	Caffeine intervention	Days to caffeine	Dose	Neurodevelopmental outcome
CAP trial 18–21-month follow-up^47^	RCT follow-up	1869	VLBW (500–1250 g)	Caffeine vs Placebo	Within 10 days	20 mg/kg LD, 5–10 mg/kg/day MD	Caffeine group had reduced rates of cerebral palsy, cognitive delay, and severe retinopathy.
CAP trial 5-year follow-up^17^	RCT follow-up	1640	VLBW (500–1250 g)	Caffeine vs Placebo	Within 10 days	20 mg/kg LD, 5–10 mg/kg/day MD	Rates of cerebral palsy and cognitive delay were no longer significantly different between groups. The caffeine group had less severe impairment in gross motor skills, improved scores on the Movement Assessment Battery for Children and better motor coordination and visual perception. With further analysis, caffeine was found to have reduced rates of DCD^116^.
CAP trial 11-year follow-up^48^	RCT follow-up	1202	VLBW (500–1250 g)	Caffeine vs Placebo	Within 10 days	20 mg/kg LD, 5–10 mg/kg/day MD	The caffeine group continued to have less motor impairment than the placebo group. Self-reported quality of life was similar between groups^117^.
Latte Trial^109^	RCT in progress	132	34–36 weeks GA	4 different dose groups of caffeine vs placebo	Within 96 h	10, 20, 30, 40 mg/kg LD 5, 10, 15, 20 mg/kg/day MD	Study currently in progress and will assess neurodevelopmental outcomes at 2.5 years by comparing the 20 mg/kg dose group to the placebo arm.
Effect of early caffeine on neurodevelopmental outcome of very low birth weight newborns^74^	Retrospective observation study	160	VLBW (<1500 g) and ≤32 weeks GA	Early vs late caffeine initiation vs no caffeine	Early: ≤48 h Late: >3 day	Standard therapeutic doses	Compared to infants in the late and no caffeine group, early dosing of caffeine was associated with a significantly higher Bayley composite, cognitive, language and motor scores at 12–22 months corrected age.
Early caffeine administration and neurodevelopmental outcomes in preterm infants^110^	Retrospective observational study	2108	<29 weeks GA	Early vs late caffeine initiation	Early: ≤2 days Late: >2 days	Standard therapeutic doses	Significant neurodevelopmental impairment and odds of a low cognitive score were lower in the early caffeine group compared to the late caffeine group.
A pilot randomised trial of high-dose caffeine therapy in preterm infants^45^	Pilot RCT	74	≤30 weeks GA	High dose vs standard dose	Within 24 h	High: 80 mg/kg total over 36 h Standard: 20 mg/kg total over 36 h	Infants in the high-dose caffeine group had an increased incidence of CBH, more hypertonicity, and more deviant neurological signs at term equivalent age. No differences in neurodevelopmental outcomes at 2 years.
5-year follow-up of pilot study^111^	RCT follow-up	74	≤30 weeks GA	High dose vs standard dose	Within 24 h	High: 80 mg/kg total over 36 h Standard: 20 mg/kg total over 36 h	No significant differences between groups for socioemotional and neurodevelopmental scores at 5 years.
Early high-dose caffeine citrate for extremely preterm infants: neonatal and neurodevelopmental outcomes^108^	Retrospective observation study	218	<28 weeks GA	High vs standard dose	Within 36 h	High: 80 mg/kg LD Standard: 20 mg/kg LD. MD: 20 mg/kg	There was no difference in rates of CBH or neurodevelopmental outcomes between the standard and high-dose groups.
Caffeine citrate for very preterm infants: effects on development, temperament and behaviour^112^	RCT	287	<30 weeks GA	High vs standard dose	Not recorded	High: 80 mg/kg LD, 20 mg/kg/day MD Low: 20 mg/kg/day LD, 5 mg/kg/day MD	Not associated with significant changes in development, temperament or behaviour at 1 and 2 years of age.

*RCT* randomised controlled trial, *VLBW* very low birth weight, *GA* gestational age, *VLBW* very low birth weight, *LD* loading dose, *MD* maintenance dose, *CBH* cerebellar haemorrhage.

### Putative mechanisms of caffeine as a neuroprotectant

Caffeine has a wide range of targets and signalling pathways, making it difficult to determine its exact molecular and cellular mechanisms of neuroprotection. Caffeine primarily acts through antagonising the adenosine receptors A1R and A2AR.^40^ Other cellular mechanisms through which caffeine acts include: inhibiting phosphodiesterase, mobilising intracellular calcium, interfering with GABA receptors and enhancing N-methyl-D-aspartic acid (NMDA) excitatory postsynaptic potentials.^18^ However, millimolar concentrations of caffeine are used in these studies, therefore unlikely therapeutic caffeine levels and likely not relevant to neonatal neuroprotective mechanisms. Caffeine also has effects beyond the brain, increasing minute ventilation, CO_2_ sensitivity, central nervous system respiratory drive, cerebral autoregulation and closure of patent ductus arteriosus.^11,38,39^

**Fig. 1:**
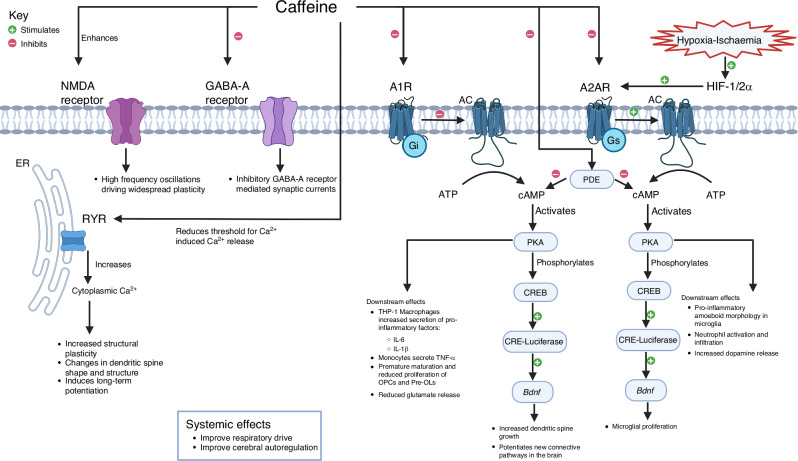
Caffeine’s cell signalling mechanisms of neuroprotection. NMDA N-methyl-D-aspartic acid, GABA gamma-aminobutyric acid, A1R: adenosine-1 receptor, A2AR adenosine-2A receptor, HIF-1/2α hypoxia-inducible factor 1/2α, AC adenylate cyclase, ER endoplasmic reticulum, RYR ryanodine receptor, ATP adenosine triphosphate, cAMP cyclic AMP, PDE phosphodiesterase, PKA protein kinase A, IL interleukin, TNF-α tumour necrosis factor α, OPC oligodendrocyte precursor cell, pre-OL pre-oligodendrocyte. Created in BioRender.

Caffeine has a similar structure to the agonist for the A1R and A2ARs, adenosine, allowing it to bind and block the action of these receptors. Adenosine is mostly produced by neurons in the brain and increases rapidly during periods of hypoxia or ischaemia, causing activation of adenosine receptors.^55,56^ The A1R is mostly concentrated in the hippocampus, cerebral cortex and cerebellum, whereas A2ARs are mostly found in the striatum, globus pallidus, and in astrocytes, microglia and blood vessels. The A1R and A2ARs inhibit and promote AC, respectively, influencing the production of cyclic AMP (cAMP) and affecting activation of protein kinase A (PKA).^40^ Through altering the activity of PKA, these receptors are able to influence the phosphorylation of downstream transcription factors like CREB, which can promote the transcription of factors associated with neuronal survival.^18^ Through its antagonism of A1R and A2AR, and the subsequent effect on cAMP production, caffeine is able to facilitate a host of downstream processes, which are discussed in this paper and summarised in Fig. 1.

Caffeine’s role as a neuroprotectant received much interest following the publication of the CAP trial, which found evidence that caffeine confers long-term neurodevelopmental benefits to LBW infants.^17,49,50^ This compound has both direct and indirect effects on neurodevelopmental outcomes in preterm infants, which this review attempts to analyse. Four main mechanisms have been identified through which caffeine could have its direct neuroprotective benefits: as a promoter of myelin production, a modulator of inflammation, an anti-apoptotic agent, and an agent of neural remodelling. Before discussing these processes, we first consider caffeine’s indirect effects on neurodevelopment.

### Caffeine’s indirect mechanisms of neuroprotection

The ability of caffeine to relieve hypoxic respiratory depression and improve cerebral autoregulation is instrumental to its indirect neuroprotective abilities. Caffeine plays an important role in improving the regulation of systemic blood pressure and cerebral haemodynamics in premature infants. Cerebral autoregulation is a mechanism where cerebral blood flow is maintained at a relatively constant level over a range of different perfusion pressures. Impairment of this system is common in preterm infants and predisposes them to intraventricular haemorrhage and its associated poor outcomes.^57,58^ A study of 30 preterm infants observed a reduced correlation between mean arterial blood pressure and tissue oxygenation index signals following caffeine administration. This suggested that caffeine had enhanced the cerebral autoregulation of those infants.^59^ This finding is supported by the reduced need for medical or surgical management of patent ductus arteriosus in caffeine-treated LBW infants.^15^ This suggests that caffeine reduces fluctuations in systemic blood pressure and stabilises cerebral blood flow.^39^ This is further supported by a study of very preterm infants that found an association between early administration of caffeine and improvements in blood pressure control and systemic blood flow.^60^ Previously, there were concerns that caffeine altered cerebral haemodynamics in preterm infants, but one study has shown that significant reductions in cerebral blood flow do not occur.^61^ In this study, the preterm infants received one dose of caffeine 2.5 mg/kg/day; therefore, it is important to examine these outcomes following larger frequent doses of caffeine. Yet, it is possible that caffeine’s circulatory benefits stem from its reduction of mechanical ventilation rates, as asynchronous spontaneous breathing during ventilation has been associated with fluctuations in arterial blood pressure.^62^

In addition to its circulatory benefits, caffeine also reduces hypoxic respiratory depression and the need for oxygen supplementation in preterm infants. The neonatal brain is vulnerable to hypoxic insult, and without adequate respiratory control, infants can experience intermittent-hypoxic events. This results in the need for oxygen supplementation, which can convert relative hypoxia into hyperoxia.^63^ Both hypoxia and hyperoxia increase the risk of multiple adverse events in the development of white matter, discussed later in this review.^64^ Increasing exposure of infants to apnoeic events and intermittent hypoxia has been associated with poor neurodevelopmental outcomes.^65^ Thus, by addressing AOP, caffeine would be expected to have neuroprotective effects. In an 18–21-month follow-up of the CAP trial, caffeine was found to have improved survival without neurodevelopmental disability.^49^ Infants in the placebo group required mechanical ventilation and supplemental oxygen for a week more, on average, than infants in the caffeine group. This prolonged use of ventilation in LBW infants has been associated with worse neurodevelopmental outcomes.^66^ Post hoc analysis suggested that the earlier discontinuation of positive airway pressure in infants assigned to caffeine was the most important intermediate variable—explaining almost half of caffeine’s effect on rates of survival without neurodevelopmental disability. Additionally, ventilator-induced lung injury predisposes infants towards developing BPD, and exposure to supplemental oxygen increases the risk of developing retinopathy of prematurity.^66,67^ These two conditions are known risk factors for poor neurodevelopmental outcomes.^66,68^ Thus, caffeine’s ability to improve hypoxic respiratory depression and circulatory control in preterm infants could be neuroprotective. Although these indirect mechanisms are important to consider, they do not explain caffeine’s full effect on outcomes in human trials^49^ or the improvements it provides to cerebral white matter development.^69^ Therefore, caffeine has molecular mechanisms of neuroprotection that must be explored.

### Caffeine as a promoter of myelin production

Oligodendrocytes are the cells responsible for myelination in the brain and develop from oligodendrocyte precursor cells (OPCs) to pre-oligodendrocytes (pre-OLs), to immature and then mature oligodendrocytes.^70^ Pre-OLs are responsible for the myelination of white matter axons from 30 weeks of gestation to post-term. This process is critical for both axonal differentiation and function, making pre-OLs critical for early cerebral cortical development.^34^ These cells are susceptible to hypoxia, ischaemia, and inflammation, with death occurring via multiple mechanisms.^71^ This results in hypomyelination and reduced brain volumes, like that seen in dysmaturation, increasing the risk of impaired development.^31,34^ Caffeine, which treats both hypoxia and inhibits the A1 receptor, is a promising substance to protect against these processes. The A1 adenosine receptor is present on oligodendrocyte cells and, when stimulated with adenosine, accelerates maturation of OPCs.^70,72^ Hypoxia also causes a similar effect in pre-OLs and OPCs, which undergo premature maturation and reduced proliferation in low oxygen conditions.^73^ Thus, hypoxia and activation of the A1 receptor both contribute to the premature differentiation of OPCs and pre-OLs, which is thought to lead to a reduction in the number of oligodendrocytes in the preterm brain.^70^ It has been hypothesised that this occurs through a hypoxia-triggered rise in adenosine levels, leading to excessive activation of A1R and an imbalance in calcium signalling that results in calcium overload in developing oligodendrocytes, resulting in poor differentiation and impaired myelination.^18^ Although this theory was proposed in a recent review,^18^ the supporting primary source has since been retracted.^74^ An alternative theory many studies have suggested is that hypoxia induces an oligodendrocyte differentiation arrest, potentially through hypoxia-inducible factor (HIF)1/2α stabilisation and inhibiting myelination.^75–77^ As mentioned below, HIF induces adenosine target receptors, and this could be a downstream effect. The role of the A1R and HIF as inhibitors of myelination is mostly speculative and requires further research. Evidence from animal studies suggests that caffeine promotes improved myelination. Studies conducted on neonatal rats found that caffeine treatment increased myelin hyperplasia. Hypoxic mice treated with caffeine saw lower rates of ventriculomegaly, increased myelination, and more immature oligodendrocytes in the caffeine group. In addition to this, the myelin in the caffeine group was better arranged and oriented compared to the hypoxic group who was not treated.^78^ This corroborates findings that myelin basic protein, which supports the structure of myelin, is reduced in rats with hypoxic brain damage and preserved in caffeine-treated groups.^64,78^ Importantly, the landmark CAP trial had a subgroup of 70 preterm infants who underwent magnetic resonance imaging (MRI) at term corrected age. This study found changes that were consistent with improvements in the microstructural development of white matter in the caffeine-treated group.^69^ Caffeine treatment was associated with MRI changes that represented more mature white tissue.^69,79^ This strongly suggests that caffeine may have a role in promoting myelin formation and improving oligodendrocyte development. Whether this role is a direct effect of cell autonomous mechanisms, or a consequence of caffeine’s indirect effects on ischaemia and hypoxia has yet to be thoroughly investigated through in vitro and conditional knockout studies, and this remains a significant knowledge gap.

### Caffeine as a modulator of inflammation

As oxidative stress and inflammation are thought to be the main causes of white matter disease in premature infants, it becomes important to establish whether caffeine has any influence over these factors.^64^ Preterm infants are subject to multiple factors that increase the risk of inflammation and oxidative stress. Changes in oxygen exposure that occur with birth lead to a period of hyperoxia with inadequate respiratory control, leading the intermittent-hypoxic events. For preterm infants, who possess an underdeveloped antioxidant system and lack alveolar surfactant, this environment of hyperoxia and hypoxia leads to oxidative stress.^63^ The idea that a connection may exist between adenosine levels, hypoxia, inflammation following preterm birth, and imbalanced inflammatory signalling is not new.^56^ Studies on LBW infants report an increased neurodevelopmental benefit from early caffeine treatment in infants with chorioamnionitis compared to placebo and late treatment.^80^ It has been proposed that caffeine has anti-inflammatory properties, which may negate adverse neurodevelopmental effects from inflammation caused by chorioamnionitis.^80,81^ Multiple mechanisms exist through which this could be achieved: caffeine’s modulation of adenosine receptors, cytokine release, microglia function, and antioxidant properties.

### Adenosine receptors as modulators of immune function

Adenosine, an agonist for A1R and A2ARs, accumulates rapidly in environments of inflammation, hypoxia and ischaemia. Hypoxia and inflammation also cause the increased action of HIF, which induces A2AR expression. Thus, in situations of hypoxia or inflammation, the expression of adenosine target receptors and agonists is amplified.^55,70^ Interestingly, caffeine has been found to suppress the accumulation of HIF-1α in primary neuronal cultures prepared from rats and promote neuron cell viability in neonatal mice exposed to hypoxic conditions.^82^ A2AR is expressed on inflammatory cells and mediates inhibitory signals by increasing intracellular cAMP upon activation.^40^ Although activation of A2AR on these cells results in generally anti-inflammatory effects, this varies depending on the cell type activated.^83,84^ In vitro studies show that A2ARs present on neutrophils cause infiltration when activated.^83,84^ Notably, neutrophils are known contributors to brain injury in neonatal rat hypoxic-ischaemic (HI) models, with neutropenia causing reduced brain swelling.^85^ Thus, caffeine may reduce brain swelling in neonatal HI injury through antagonising A2ARs on neutrophils. The A1R has similarly been described in the literature to either increase inflammation or reduce it in some cases.^85^ It is hypothesised that this is because, in conditions of high serum adenosine, like that seen in HI environments, the A1R deviates from its normal function and increases calcium influx, causing cell death.^85^ In vitro activation of the A1R in THP-1 macrophages increases the secretion of pro-inflammatory factors interleukin (IL)-6 and IL-1b, suggesting that antagonism would be anti-inflammatory.^86^ Meanwhile, agonism of the A1R has been shown to protect from inflammation in ischaemic injury models.^87^ It is possible that these conflicting actions are the result of receptors being analysed across different models, for example, inhibiting the A1R is neuroprotective for neonatal rodents but increases brain vulnerability in embryonic rodents.^70^ Overall, for both the A1R and A2ARs data are conflicting and suggest that the antagonism of these receptors could be either pro- or anti-inflammatory depending on the situation and location of the receptor.^70,85^ The role of these receptors in modulating inflammation is currently poorly understood within the literature and requires further study.

### The effect of caffeine on cytokine release

It is well established that caffeine reduces markers of inflammation.^88^ Many studies have shown that the administration of caffeine decreases cytokine release and other pro-inflammatory markers in rat, mouse and ovine models of neonatal HI injury^89–93^ with reduced lesions in brain matter^92,93^ and improvements in neurodevelopmental outcomes.^90,92,93^ Specifically, in neonatal rats, caffeine reduces the pro-inflammatory cytokines IL-1β and tumour necrosis factor (TNF)-α^90,91^ and promotes transcription of the anti-inflammatory factors IL-10 and transforming growth factor-β.^90^ A human study on the effect of caffeine as an antagonist of A1R in cord blood monocytes found that caffeine reduced production of pre-transcriptional TNF-α, inflammation and cell apoptosis.^94^ An observational prospective study of caffeine levels and cytokine profiles in peripheral blood and tracheal aspirates of preterm infants,^95^ reported that at therapeutic levels (10–20 μg/mL) caffeine was associated with a reduction in the pro-inflammatory cytokines IL-6 and TNF-α and an increase in IL-10 anti-inflammatory cytokine levels. Interestingly, caffeine levels outside of therapeutic range (≥20 μg/mL) were associated with a pro-inflammatory profile. This suggests that caffeine promotes an anti-inflammatory cytokine profile in human models when administered within therapeutic ranges.

### The role of microglia

Microglia are an important component of the brain’s neuroinflammatory response, clearing pathogens and responding to cellular debris. They are distributed throughout the brain from 20 to 35 weeks of gestation and are prominent during this critical period of neural maturation. They are generally understood to have two forms, an activated pro-inflammatory form (M1) and an anti-inflammatory form (M2). In their pro-inflammatory form, microglia can be destructive, importantly to pre-OLs, through generating free radicals, secreting cytokines (IL-1β, IL-6, iNOS, and TNF-α) and enhancing excitotoxicity. If pro-inflammatory insults occur during this key period of maturation, such as the intermittent-hypoxic events that preterm infants may experience, then diversion of microglia into the M1 pro-inflammatory state could cause neural damage.^34^ The activation of the A2AR changes microglia to amoebic morphology in in vitro studies of human microglia.^96^ In rat models, activation has also been shown to increase expression of M1 pro-inflammatory cytokines.^97^ A2AR antagonists inhibit microglial activation; thus, caffeine could theoretically reduce neuroinflammation through its antagonism of the A2AR.^18,98^

Evidence from animal studies supports the notion that caffeine prevents microglial activation, reducing neuroinflammation. Rats exposed to HI conditions were treated with caffeine and found to have reduced activation of microglia 1ba-1, inhibited polarisation of M1 microglia and increased the polarisation of M2 microglia with improved protection of myelin, reduced ventriculomegaly and long-term cognitive benefits.^90^ Another study similarly exposed neonatal mice to HI conditions and noted that the caffeine group had a significantly decreased number of amoeboid microglia and a reduced area covered by astrogliosis, with improved neurodevelopmental outcomes.^92^ Caffeine treatment in rats with HI saw protective benefits with reduced neuronal cell death; however, microglial activation was only reduced in male rats. This was hypothesised to be the result of the later maturation of microglia in males, which makes them more vulnerable to inflammation than females, whose microglia are typically more protective in nature. This study was interesting in that it suggested that mechanisms of action may be different between the sexes.^99^ Finally, caffeine treatment in mice with intraventricular haemorrhage saw a reduced microglial load and a reduction in neuroinflammation.^100,101^ Thus, animal studies suggest that caffeine may result in neurodevelopmental benefits through reducing the number of microglia and their activation to create an anti-inflammatory environment that is beneficial to oligodendrocyte development and early neural development. Although human in vitro studies suggest that caffeine achieves this effect through antagonism of the A2AR, further evidence is required to directly link caffeine to microglial changes in a controlled in vitro environment.

### Caffeine as an antioxidant

Oxidative stress and free radical damage are also important causes of neurotoxic damage in preterm infants.^64^ Neurons contain low levels of exogenous antioxidants, making the brain vulnerable to oxidative stress generated by HI events.^102^ In addition to this, oxidative stress has been found to selectively target pre-OLs, the depletion of which is a notable feature in white matter injury.^103^ Caffeine has exhibited antioxidant activity in vitro, inhibiting lipid peroxidation and reducing the activity of reactive species.^104^ One study that exposed rats to hyperoxia found that caffeine reduced oxidative stress markers and increased the anti-oxidative response.^89^ Another study examined neonatal rats in conditions of normoxia and also found that caffeine reduced oxidative stress.^105^ Further supporting these findings, a recent study exposed neonatal rats to HI before treating them with caffeine and found a reduction in oxidative stress markers, NOX-1 and NOX-2, in addition to a decrease in pro-inflammatory cytokines.^91^ Hence, there is evidence to suggest that caffeine protects against oxidative stress. Through its protection against oxidative stress and neuroinflammation, it could be expected that caffeine would also reduce apoptosis, synaptic defects, and neuronal cell death.

### Caffeine as an anti-apoptotic agent

Part of caffeine’s neuroprotective effects may stem from its anti-apoptotic activity. Neonates exposed to hyperoxia have increased apoptotic neuronal cell death, while caffeine has been found to protect against neuronal damage and cell death under those same conditions.^106,107^ Multiple studies have shown that caffeine reduces the number of pro-apoptotic effector cells^89,92,105,106,108^ in neonatal rodent models of HI injury with improvements in neurodevelopmental outcomes.^92^ Some studies have highlighted the various ways in which caffeine achieves this. Caffeine has been found to reduce the pro-apoptotic effectors caspase-3, poly(ADP-ribose)polymerase 1 (PARP-1), and apoptosis-inducing factor (AIF), and to reduce extracellular matrix degeneration and inhibit metalloproteinases in neonatal rats.^89^ An in vitro study using cortical neurons from littermate embryos found that caffeine inhibited hydrogen peroxide-induced apoptotic neuronal death. Inhibition of the downregulation of the anti-apoptotic proteins Bcl-2 and Bcl-X_L_ was seen, and inhibition of the pro-apoptotic proteins caspase-3 and Pro-PARP.^109^

While evidence suggests that caffeine reduces apoptosis, it is important to consider the events that trigger apoptosis. One theory is that caffeine’s protection against oxidative stress and neuroinflammation may lead to reduced neuronal cell damage and apoptosis, decreasing white matter loss and brain lesions.^18,64^ Another theory relates to the temporary increase in cortical electrical activity seen in preterm infants following caffeine administration.^110,111^ Low levels of neural activity may predispose neonatal neurons to undergo apoptosis; thus, through increasing brain activity, caffeine would boost intrinsic antioxidant activities and prevent this.^110^ Ultimately, it is well established that caffeine inhibits apoptosis, an important component of its neuroprotective mechanisms, which sees increased preservation of neuronal architecture and function. Further research is required to determine exactly how caffeine achieves this effect.

### Caffeine as an agent of neural remodelling

The idea that caffeine may promote enhanced synaptic formation and neural plasticity is one of the more recent neuroprotective mechanisms proposed.^16^ Caffeine’s role as an A1R/A2AR antagonist allows it to affect synaptic transmission through increasing and decreasing the release of neurotransmitters such as glutamate and dopamine, respectively.^18^ At non-therapeutic doses, caffeine also enhances NMDA receptor activities, driving widespread neuronal plasticity, reduces GABA-A receptor-mediated inhibitory synaptic activity, and influences intracellular calcium signalling pathways, inducing increased structural plasticity, changes in dendrite spine size and shape, and long-term potentiation.^16,41^

CREB is a transcription factor that is phosphorylated in response to mitogens, neurotrophins and other neuronal growth factors. It is involved in the transcription of genes that are essential for the development and function of the nervous system. Activation of CREB has been associated with neuronal maintenance and sparing following ischaemic injury. Loss of the CREB gene is associated with progressive neurodegeneration.^112^ An important gene that CREB transcribes is the *Bdnf* gene, which codes for brain-derived neurotrophic factor (BDNF). BDNF promotes neuron survival, regulates synaptogenesis and synaptic plasticity mechanisms involved in learning and memory in the adult central nervous system.^113^ BDNF levels are reduced in preterm infants, compared to term infants, with lower levels correlating with worse neurodevelopmental outcomes. BDNF plays a significant role in both the nervous and respiratory systems, with downregulation of BDNF occurring in damage models of these immature systems.^63^

A study using developing mouse cortical neurons, exposing them to caffeine and observing the effect on CREB-dependent gene transcription, found that the CREB binding protein was stimulated by caffeine, exhibiting a bell-shaped dose-response curve. Caffeine exposure increased gene transcripts from CREB target genes, with an increase in CRE-luciferase activity and *Bdnf* expression.^114^ This activity increases dendritic spine growth, alters neural networks, changes the morphology of synapses and potentiates new connective pathways in the brain.^16^ A 2006 study administered caffeine to neonatal rats for the first twelve days of life and found that animals that had been administered caffeine had longer dendrite length in their pyramidal neurons in the third layer of the prefrontal cortex. The overall volume of the caffeine group’s prefrontal cortex was increased, and these effects persisted until puberty.^115^ Furthermore, a study in a neonatal rat model found that caffeine attenuated hyperoxia-mediated reductions in the expression of transcription factor mRNA that was important to the process of neurogenesis.^106^ Interestingly, A2AR antagonism has been associated with reduced BNDF release and microglial proliferation in murine cell cultures, which would seemingly contradict the previous findings, and this requires further investigation.^98^ Preterm infants are at particular risk for white matter disease, which sees the damage of axon growth, synaptogenesis and neurogenesis, making it highly important that pharmacological treatments in this population promote neural remodelling.^64^

### Caffeine as a neuroprotectant in preterm infants

Clinical trials of preterm infants treated with caffeine have yielded a range of neurodevelopmental outcomes.^17,46,49,50,80,116–120^ (Table 1) A broad analysis of these findings suggests that caffeine confers long-term neurodevelopmental improvements in motor abilities but does not confer benefits in any other neurodevelopmental domains.^121^ Evidence tentatively suggests that early administration of caffeine improves neurodevelopmental outcomes, with dose changes having no significant impact. Overall, few high-quality studies exist to support changes in current practices of administering caffeine for its use as a neuroprotectant.

## Conclusions

This narrative review analysed the role of caffeine as a neuroprotectant in preterm infants. The landmark CAP trial established, through a series of follow-up studies, that caffeine may provide short-term neurodevelopmental benefits that attenuate with time. The only persisting neurodevelopmental effect that caffeine appears to provide is in protecting long-term motor function in preterm infants. Few studies have analysed ways in which the administration of caffeine can impact neurodevelopmental outcomes, and this requires further analysis through high-quality studies. The putative mechanisms of neuroprotection offered by caffeine were evaluated. The hypothesis that caffeine directly confers neuroprotective benefits through altering white matter structures, reducing inflammation, preventing cell death and conferring neuromodulatory benefits has increasingly more evidence. However, it remains difficult to distinguish caffeine’s indirect effects on neurodevelopment from its direct effects, as most evidence has been collected from in vivo animal studies. Further discussion and research are required to better understand these mechanisms and the way in which they could impact neurodevelopment. As neuroprotective treatments become increasingly important in managing preterm births, the role of caffeine as a neuroprotectant and its modes of action will only increase.
